# Tracing Back the Evolutionary Route of Enteroinvasive *Escherichia coli* (EIEC) and *Shigella* Through the Example of the Highly Pathogenic O96:H19 EIEC Clone

**DOI:** 10.3389/fcimb.2020.00260

**Published:** 2020-06-03

**Authors:** Valeria Michelacci, Rosangela Tozzoli, Silvia Arancia, Alfio D'Angelo, Arianna Boni, Arnold Knijn, Gianni Prosseda, David R. Greig, Claire Jenkins, Teresa Camou, Alfredo Sirok, Armando Navarro, Felipe Schelotto, Gustavo Varela, Stefano Morabito

**Affiliations:** ^1^Department of Food Safety, Nutrition and Veterinary Public Health, Istituto Superiore di Sanità, Rome, Italy; ^2^Department of Biology and Biotechnology “Charles Darwin”, Università Sapienza di Roma, Rome, Italy; ^3^Gastrointestinal Bacteria Reference Unit (GBRU), Public Health England, E. coli, Shigella, Yersinia and Vibrio Reference Service, National Infection Service, London, United Kingdom; ^4^Departamento de Laboratorios, Ministerio de Salud Pública, Montevideo, Uruguay; ^5^Public Health Department, Medicine Faculty, Universidad Nacional Autónoma de Mexico (UNAM), Mexico City, Mexico; ^6^Departamento de Bacteriología y Virología, Facultad de Medicina, Instituto de Higiene, Universidad de la República, Montevideo, Uruguay

**Keywords:** EIEC, phylogenomics, conjugation, evolution, emerging clones

## Abstract

Enteroinvasive *Escherichia coli* (EIEC) cause intestinal illness through the same pathogenic mechanism used by *Shigella* spp. The latter species can be typed through genomic and phenotypic methods used for *E. coli* and have been proposed for reclassification within *E. coli* species. Recently the first appearance of a highly pathogenic EIEC O96:H19 was described in Europe as the causative agent of two large outbreaks that occurred in Italy and in the United Kingdom. In contrast to *Shigella* spp and to the majority of EIEC strains, EIEC O96:H19 fermented lactose, lacked pathoadaptive mutations, and showed good fitness in extracellular environment, similarly to non-pathogenic *E. coli*, suggesting they have emerged following acquisition of the invasion plasmid by a non-pathogenic *E. coli*. Here we describe the whole genome comparison of two EIEC O96:H19 strains isolated from severe cases of diarrhea in Uruguay in 2014 with the sequences of EIEC O96:H19 available in the public domain. The phylogenetic comparison grouped all the O96:H19 strains in a single cluster, while reference EIEC strains branched into different clades with *Shigella* strains occupying apical positions. The comparison of the virulence plasmids showed the presence of a complete conjugation region in at least one O96:H19 EIEC. Reverse Transcriptase Real Time PCR experiments confirmed in this strain the expression of the pilin-encoding gene and conjugation experiments suggested its ability to mobilize an accessory plasmid in a recipient strain. Noteworthy, the *tra* region was comprised between two reversely oriented IS*600* elements, which were also found as remnants in another EIEC O96:H19 plasmid lacking the *tra locus*. We hypothesize that an IS-mediated recombination mechanism may have caused the loss of the conjugation region commonly observed in EIEC and *Shigella* virulence plasmids. The results of this study support the hypothesis of EIEC originating from non-pathogenic *E. coli* through the acquisition of the virulence plasmid via conjugation. Remarkably, this study showed the ability of a circulating EIEC strain to mobilize plasmids through conjugation, suggesting a mechanism for the emergence of novel EIEC clones.

## Introduction

Enteroinvasive *Escherichia coli* (EIEC) cause disease in humans, characterized by abdominal cramps, bloody and mucous diarrhea (van den Beld and Reubsaet, 2012). EIEC are able to invade and multiply in the human colonic epithelial cells analogously to the mechanism used by *Shigella* spp (Nataro and Kaper, 1998; Lan et al., 2004), making it difficult to differentiate between the disease caused by the two microorganisms. The main virulence genes of EIEC and *Shigella* are harbored on a large virulence plasmid termed pINV. These include the *mxi* and *spa* genes encoding a type three secretion system (T3SS) as well as the *ipaB, ipaC, ipaD*, and *icsA* genes encoding effectors necessary to invade and disseminate into the host cells (Pasqua et al., 2017). The transmission of the infection occurs *via* the fecal-oral route and the incidence of EIEC and *Shigella* infections is higher in geographical areas where there is less or no access to safe drinking water, health services, or electricity. However, infection may also occur by the ingestion of contaminated food or water. During the last decade an increase in the number of cases of EIEC infections has been observed in Europe, with two large outbreaks, suspected to be linked to the consumption of contaminated food, occurred in Italy and in the United Kingdom between 2012 (Escher et al., 2014) and 2014 (Newitt et al., 2016). The strains responsible for both the outbreaks belonged to the O96:H19 serotype and sequence type 99 (ST-99), which had never been described as EIEC before 2012. A third isolate sharing the same characteristics was also identified as the cause of a sporadic case of EIEC infection occurred in Spain in 2013, confirming the circulation of such clone in Europe (Michelacci et al., 2016).

The genomic characterization of these three strains enabled the detection of an IncFII plasmid larger than 200 kbp, resembling the invasion plasmid of EIEC and *Shigella* and harboring the virulence genes essential for intracellular localization and spread, but in a genomic background different from that of reference EIEC and *Shigella* strains (Michelacci et al., 2016). This led to the hypothesis that O96:H19 EIEC clone might have emerged after the acquisition of the virulence plasmid by an *E. coli* with the phenotypic and biochemical properties of a commensal *E. coli* strain (Michelacci et al., 2016).

In the present study, we report the description of two O96:H19 EIEC strains isolated from two patients in Uruguay in 2014 and describe the genomic comparison of their chromosome and plasmids with those of the O96:H19 EIEC strains isolated in Europe. Our data support the hypothesis that this EIEC clone may have emerged and spread thanks to pINV mobilization through conjugation and provide evidence that at least one of the EIEC O96:H19 studied possessed a complete conjugation region in the pINV and displayed a functional plasmid mobilization machinery.

## Materials and Methods

### Bacterial Strains and Genomes

Two EIEC strains from Uruguay were included in this study: the strains V48 and V73 were isolated from fecal samples of an 18 month-old girl with bloody diarrhea and of a 14 year-old girl with fever, vomiting, abdominal pain, bloody diarrhea and shock in April and October 2014, respectively. The latter strain was isolated form a case part of a foodborne outbreak and there was no epidemiological link between the two cases (Peirano et al., 2018).

Genomes of EIEC O96:H19 available in the public domain were included in the comparative analyses. These included those of strains EF432, 152661 and CNM-2113/13, isolated during EIEC outbreaks occurring in Italy (Escher et al., 2014; Michelacci et al., 2016) and the UK (Michelacci et al., 2016; Newitt et al., 2016) and from a sporadic case in Spain, respectively (Michelacci et al., 2016), and the genomes of four other EIEC O96:H19 strains described in a previous study on EIEC circulating in the United Kingdom (details in Supplementary Table 1) (Cowley et al., 2018). The presence of the *ipaH* genetic marker of EIEC and *Shigella* was confirmed by PCR (Luscher and Altwegg, 1994) or *in silico* in all the *E. coli* strains included in the study.

The *E. coli* K12 strain CSH26 Nal^r^ (Sorensen et al., 2003), showing resistance to nalidixic acid and sensitivity to streptomycin and sulfamethoxazole, was used as recipient strain in conjugation experiments with donor EF432 strain.

Genomes from reference EIEC and *Shigella* strains as well as from EIEC strains recently described to circulate in the UK and belonging to different serotypes (Cowley et al., 2018) were retrieved from international databases and analyzed to give context to the phylogenetic comparison (details in Supplementary Table 1).

### DNA Extraction and Sequencing

The total DNA of the strains V48, V73, and CNM-2113/13 was extracted from two ml of overnight bacterial cultures with the GRS Genomic DNA kit (GRISP, Porto, Portugal) and sequenced on an Ion Torrent S5 platform (Life Technologies, Carlsbad, USA). In detail, 400 bp fragments libraries were prepared by using the NEBNext® Fast DNA Fragmentation & Library Prep Set for Ion Torrent (New England Biolabs, Ipswich, Massachusetts, USA). The template preparation and sequencing run were performed with the ION 520/530 KIT-OT2 following the manufacturer's instructions for 400 bp DNA libraries on ION 530 chips.

The genome of the 152661 strain from the outbreak in the UK, already sequenced through Illumina technology for routine surveillance of *E. coli* and *Shigella* by Public Health England (Supplementary Table 1), was re-sequenced using a MinION system (Oxford Nanopore Technologies Ltd, Oxford, UK), producing long reads, with the aim of closing the complete sequence of the chromosome and the plasmids harbored by this strain.

In detail, total genomic DNA of 152661 strain was extracted using the Wizard Genomic DNA Purification kit (Promega, Madison, WI, USA) with significant modifications from manufacturer's instructions including no vortexing steps, double incubation and elution times and pre-chilling of 70% ethanol and 99% isopropanol before use. Library preparation was performed using the SQK-RBK004 (rapid) library preparation kit (Oxford Nanopore Technologies Ltd, Oxford, UK) according to manufacturer's instructions. The sequencing library was loaded onto a FLO-MIN106 R9.4.1 flow cell and sequenced on the MinION for 24 h.

The sequencing data generated during this project has been uploaded to the EMBL-ENA sequence database in the study with accession number PRJEB35723 and at NCBI Bioproject with Acc. No. PRJNA315192 (details in Supplementary Table 1).

### Bioinformatics Analysis

#### Basic Analyses: Trimming and Assembly

The bioinformatics analyses of Illumina and Ion Torrent data were performed through the ARIES instance of the Galaxy bioinformatics framework (https://www.iss.it/site/aries) as previously described (Michelacci et al., 2016). Briefly, FastQC was used for quality check and “FastQ Positional and Quality Trimming” (Cuccuru et al., 2014) for trimming the raw reads. The contigs were assembled from Illumina and Ion Torrent trimmed data using SPADES version 3.12.0 (Bankevich et al., 2012), followed by the tool “Filter SPAdes repeats” Galaxy Version 1.0.1 (https://github.com/phac-nml/galaxy_tools/). Default parameters were applied in the two steps.

As for the sample 152661, the data deriving from Illumina and Nanopore sequencing platforms were integrated. In detail, data produced from the MinION in a raw FAST5 format was basecalled and de-multiplexed using Albacore V2.3.3 (Oxford Nanopore Technologies, ONT) and Deepbinner v0.2.0 (Wick et al., 2018) to obtain sample-specific files in FASTQ format. Run metrics were generated using Nanoplot v1.8.1 (De Coster et al., 2018). The barcode and y-adapter were trimmed and chimeric reads split using Porechop v0.2.4^1^. Finally, the trimmed reads were filtered using Filtlong v0.1.1 with the following parameters: min length = 1,000, keep percent = 90 and target bases = 550 Mbp, to generate ~100x coverage with the longest and highest quality reads^2^.

Trimmed ONT FASTQ files were assembled using Canu v1.7 (Koren et al., 2017) and the filtered ONT FASTQ files were assembled using both Unicycler v0.4.2 (Wick et al., 2017) with the following parameters: min_fasta_length=1000, mode=normal. The assembly showing the highest N50 and lowest number of contigs, with an assembly size comprised between 5.3 and 6.0 Mbp, were used for the following analyses. Polishing of the assemblies was performed in a three-step process, firstly using Nanopolish v0.11.1 (Loman et al., 2015) with both the trimmed ONT FASTQs and FAST5s files accounting for methylation using the –methylation-aware=dcm and –min-candidate-frequency=0.5.

Secondly, Pilon v1.22 (Walker et al., 2014) was applied with Illumina FASTQ reads (Acc. No. SRR4181492) as the query dataset with the use of BWA v0.7.17 (Li and Durbin, 2010) and Samtools v1.7 (Li et al., 2009). Finally, Racon v1.2.1 (Vaser et al., 2017) also using BWA v0.7.17 (Li and Durbin, 2010) and Samtools v1.7 (Li et al., 2009) was used with the Illumina reads for two cycles to produce a final assembly for each of the samples. The chromosome from the assembly was re-circularized and closed and re-orientated to start at the *dnaA* gene, as in the reference sequence of K12 *E. coli* MG1655 strain (Acc. No. NC_000913), using the –fixstart parameter in circlator v1.5.5 (Hunt et al., 2015).

The completely assembled sequence of 152661 resulting from this process was used as such for chromosome and plasmids comparison, strain characterization and phylogenetic analysis, as described later.

#### WGS Analysis for Strain Characterization, Chromosome, and Plasmids Comparison and Phylogenetic Analysis

The WGS analyses for strain characterization and typing were performed through ARIES webserver (https://www.iss.it/site/aries). Multi Locus Sequence Typing (MLST) was inferred from the trimmed Illumina and Ion Torrent reads using the SRST2 tool (Inouye et al., 2014) and applying the scheme developed by Wirth and colleagues (Wirth et al., 2006). The “*E. coli* Serotyper” tool (Galaxy Version 1.1) was used with default parameters to interrogate the database of reference sequences for the determination of the serotypes (Joensen et al., 2015). The virulence genes typical of EIEC and *Shigella* were searched in the genome sequences as previously described Michelacci et al. (2016). Moreover, the assembled sequences of the EIEC O96:H19 strains were tested for the presence of virulence genes typical of other pathotypes of *E. coli* (Joensen et al., 2014) performing blastn analysis, using the threshold of minimum 90% of sequence identity and 80% of gene coverage.

The complete list of Accession Numbers of the sequences included in the comparison analysis is provided in Supplementary Table 1.

The Prokka tool (Seemann, 2014) was used for the functional annotation of the assembled sequences, using the *E. coli* specific gene database. Blast Ring Image Generator (BRIG) software v0.95 (Alikhan et al., 2011) was used with default parameters to compare the completely assembled sequences of the chromosome and virulence plasmid of the EIEC strains from Italy (EF432) (Pettengill et al., 2015a) used as reference sequences, with those of the other EIEC O96:H19 considered in this study. This analysis also included the completely assembled sequence of the largest plasmid of strain 152661 (Acc. No. CP046677).

The presence of pathoadaptive mutations in *cadA, cadB, cadC, speG, nadA*, and *nadB* was investigated and the sequences of *speA, speB, speC, speD, speE*, and *speF* genes verified as previously described (Michelacci et al., 2016) through the use of MAUVE software (suggested development snapshot 2015-02-26) (Rissman et al., 2009).

The detection of genetic elements involved in conjugation and the design of the maps of the closed plasmids were performed through the OriT Finder tool available online (http://202.120.12.134/oriTfinder/oriTfinder.html) with default parameters by uploading the annotated Genbank files produced with the Prokka software. MAUVE software (Rissman et al., 2009) was used for a deeper comparison of the conjugative regions in a set of plasmid sequences selected on the basis of the results of the BRIG analysis. The ISfinder webserver (https://www-is.biotoul.fr) (Siguier et al., 2006) was used to characterize and compare the insertion elements identified at the two sides of the conjugation region of pINV from strain EF432.

The core genome MLST (cgMLST) comparison was performed with the chewBBACA tool version 2.0.13 (Silva et al., 2018) with default parameters and used the database developed by EnteroBase (https://enterobase.warwick.ac.uk/) and curated in the framework of INNUENDO project, comprising the analysis of 2360 *loci* (Llarena et al., 2018) to call the alleles. The Minimum Spanning Tree was generated by analyzing the allelic matrix on the PHYLOViZ online web-based tool (Ribeiro-Goncalves et al., 2016).

### Analysis of the Expression of *traA* Pilin-Encoding Gene

RNA was extracted from one ml of overnight cultures of the strain EF432 grown at 30° and 37°C using the Norgen RNA/Protein Purification Kit (Norgen Biotek, Thorold, ON, Canada). In detail, 1 μg of extracted RNA was used for DNA removal and retro-transcription with the QuantiTect Reverse Transcription (Qiagen, Germantown, MD, USA). Two μl of the cDNA solutions were used in Real Time PCR reactions targeting *traA* gene, encoding the pilin, in 40 cycles of a two steps thermal profile (15 s at 95°C and 1 min at 55°C) using the following primers and probes: traA_FWD: AGTGATCCCGGTTGCTGTTT; traA_REV: GTACATGACTGCACCGACCA; traA_probe: CTTCTGCTGGTAAAGGCACG. The efficiency of the reaction was evaluated by using serial dilution (10^−1^,10^−2^, 10^−3^, and 10^−4^) of a 11.3 ng/μl DNA preparation purified from an overnight culture of EF432 strain as template in the same amplification run. The reactions were duplexed with reagents targeting *gapA* reference housekeeping gene, as previously described (Fitzmaurice et al., 2004). A negative control reaction was performed using non-retrotranscribed RNA. The efficiency, *R*^2^ and M values of the *traA* gene amplification were compared to those of the reaction targeting *gapA* gene.

### Bacterial Conjugation

Overnight cultures of donor strain EF432 and recipient strain CSH26 Nal^r^ were diluted 1:10 in TSB and refreshed for 1.5 h. Two ml of each culture were mixed and incubated for 3 h at 37°C without shaking. The selective marker for the recipient strain was nalidixic acid, as EF432 was proved to be susceptible to its presence in growth media. As no selection markers were found on pINV, CongoRed was used as differential additive in combination with nalidixic acid, due to its ability to identify *E. coli* strains harboring pINV plasmid as red colonies (Maurelli et al., 1984). One hundred μl of undiluted conjugation mix and 10^−1^, 10^−2^, and 10^−3^ serial dilutions were then plated and incubated at 37°C on two different media: CongoRed TSA plates supplied with 10 μg/ml of nalidixic acid and LB plates containing 10 μg/ml of nalidixic acid and 10 μg/ml of streptomycin. The colonies obtained on the latter were then streaked on LB plates containing 10 μg/ml of sulfamethoxazole for confirming the presence of the resistance plasmid of strain EF432.

## Results

### Genomic Characterization of O96:H19 EIEC Strains Isolated From Uruguay

The genomic sequences of the V48 and V73 strains isolated in Uruguay were assembled in 141 and 139 contigs with N50 values of 113,473 and 103,437 bp, respectively. *In silico* typing confirmed the O96:H19 serotype and assigned to the strains the ST-99, the same Sequence Type identified in the already described O96:H19 EIEC (Michelacci et al., 2016). The virulence gene content and the presence of pathoadaptive mutations was investigated in the strains from Uruguay and compared to the same information obtained from the whole genome sequences of all the EIEC O96:H19 strains included in the study.

The results of the identification of the virulence genes typical of EIEC and *Shigella* are shown in Supplementary Table 2. All the strains showed the same virulence gene asset already described for the O96:H19 isolated in Europe (Michelacci et al., 2016). A major exception was the strain identified with the Acc. No. SRR4786227 among the selected UK strains, which showed a peculiar asset of plasmid-borne virulence genes. In particular, the presence of the genes *ipaH, ospG, virA*, and *virF* and the absence of the region known as “entry region,” encoding the T3SS and its effectors were observed. On the other hand, the absence of *ospG* virulence gene was identified in two of these (Acc. No. SRR3578973 and SRR3578582), while in remaining one (Acc. No. SRR3578770) *ospG* gene was present, but the genes *virA* and *icsA* were lacking.

The detection of virulence genes of pathogenic *E. coli* other than EIEC allowed identifying the presence of *lpfA* and *capU* genes in all the O96:H19 strains. In particular, in the completely assembled sequences available of strains EF432 and 152661, *lpfA* gene was found on the chromosome while *capU* was detected in the virulence plasmid pINV.

The analysis of pathoadaptive mutations highlighted their absence in all the strains tested.

The comparison of the chromosomes of all the O96:H19 strains investigated is presented in Supplementary Figure 1, and showed a similar structure in all the genomes investigated. Nevertheless, some short fragments resulted only present in the sequence of the completely assembled chromosomes of strains EF432 and 152661, mainly representing regions harboring prophages and encoding tRNAs and rRNAs. The complete list of these regions differentially present in the genomes, together with the encoded functions derived from annotation is presented in Supplementary Table 3.

### Comparison of the Invasion Plasmids

**Figure 1:**
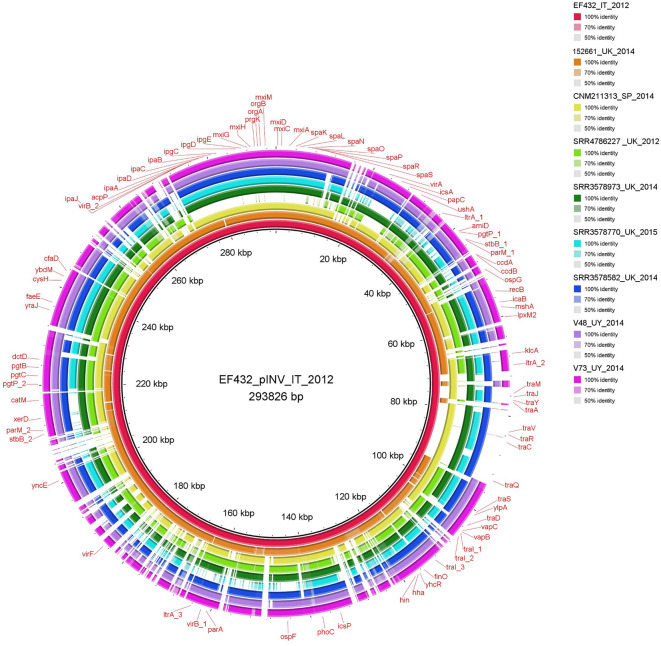
Comparison of invasion plasmids of O96:H19 EIEC strains. The completely assembled pINV from EF432 strain (inner circle, red color) responsible of the outbreak occurred in Italy in 2012 was used as reference for alignment and gene annotation.

The comparison of the sequences of invasion plasmids showed the absence of the region comprising *tra* genes in four of the nine O96:H19 strains assayed. The same region was instead present in the sequence of the invasion plasmid of the EIEC O96:H19 strain EF432 isolated in Italy (Figure 1). In detail, the sequences of pINV of the strains V48, V73, 152661 and that of the strain with the Acc. No. SRR4786227 completely lacked these genetic *loci*. Conversely, the strain from Spain CNM-2113/13 and three of the other isolates from the UK showed the presence of this *locus*, although apparently lacking some genetic fragments in the region (Figure 1).

Interestingly, a fragment longer than 30 kbp, corresponding to the “entry region” encoding the T3SS and its effectors, was absent from the plasmid of SRR4786227 strain, confirming the virulotyping results.

### Mobilization Analysis of the Invasion Plasmids of O96:H19 EIEC Strains

In order to investigate the possibility to mobilize the pINV by the EIEC O96:H19 strain harboring the complete conjugative region, a detailed search of the presence of the genetic elements involved in conjugation was performed in the sequence of the plasmids of strain EF432. In addition, a transcription assay on pilin-encoding gene *traA* and a conjugation assay among EF432 and a recipient strain were also carried out.

#### Analysis of the Presence of Conjugation-Related Genetic Elements

**Figure 2:**
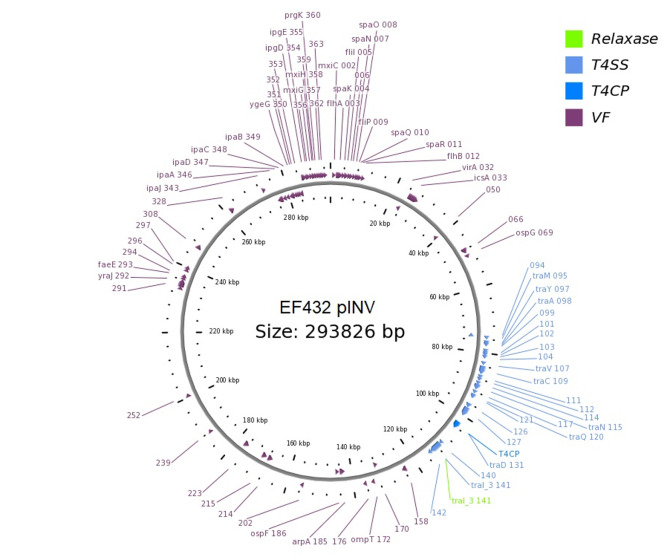
Genetic map of the pINV virulence plasmid of EF432 strain obtained through OriT Finder. The annotation includes gene prediction obtained through Prokka (Seemann, 2014) or progressive numbers for the coding sequences identified for which no gene could be called.

**Figure 3:**
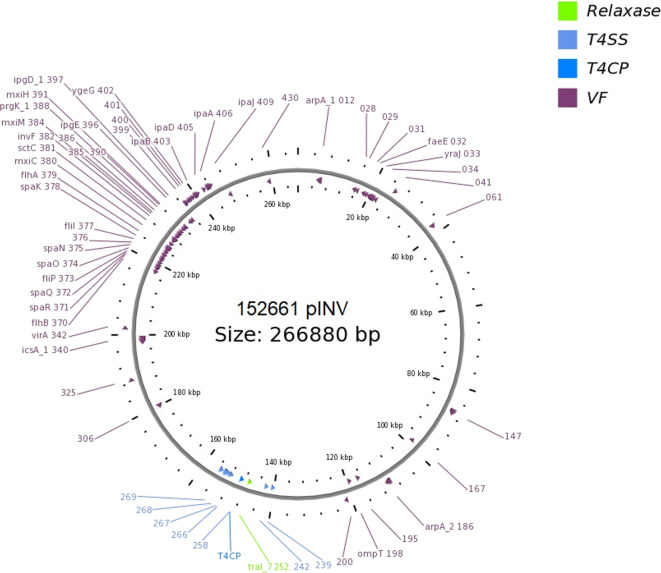
Genetic map of the pINV virulence plasmid of 152661 strain obtained through OriT Finder. The annotation includes gene prediction obtained through Prokka (Seemann, 2014) or progressive numbers for the coding sequences identified for which no gene could be called.

**Figure 4:**
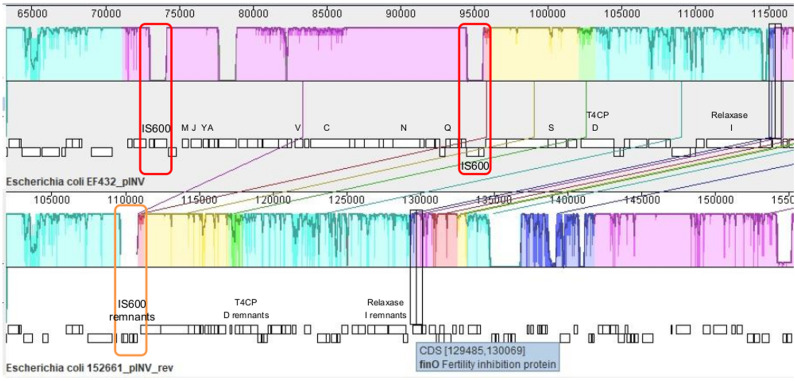
Alignment of *tra* regions in pINV of EF432 strain, used as reference, and pINV of 152661, produced with MAUVE tool (Rissman et al., 2009). Conserved segments that appear to be internally free from big genome rearrangements are visualized as colored blocks. A decrease in percentage of sequence identity is represented as a decrease in coloring. The genetic organization is represented under each sequence, with *tra* genes indicated with the corresponding letters (*traMJYAVCNQSDI*) and T4CP and Relaxase-encoding genes highlighted for their encoded function. The red boxes indicate the two identical and inverted copies of an IS*600* element in the reference, while the orange box highlights the IS*600* remnant in the corresponding region of pINV in 152661 strain.

The presence of genetic *loci* involved in plasmid transfer through conjugation was initially investigated on the closed sequences of the plasmids of strains EF432 and 152661 (Pettengill et al., 2015a). The maps of pINV plasmids from the two strains are reported in Figures 2, 3, while the maps of the accessory plasmids found in the same strains and harboring resistance genes (named pRES hereafter), are included as Supplementary Figures 2, 3. The complete list of the features involved in conjugation resulting from these analyses is reported in Table 1, showing the presence of the complete asset of conjugation-related regions only on pINV plasmid from EF432 strain (pINV_EF432_). In particular, pINV from EF432 harbored genes encoding the relaxase and type IV coupling protein (T4CP), two essential components of the ssDNA conjugation machinery, and a region of about 41 kbp encoding the Type Four Secretion System (T4SS). The pRES plasmid harbored in the same strain, possessed part of the conjugative features but lacked the T4SS gene cluster (Table 1). On the other hand, either the pINV or the pRES plasmids present in the strain 152661 lacked the origin of transfer (*oriT*), and the T4SS gene cluster region present on this pINV consisted of 16 kbp only (Table 1). A deeper analysis showed the presence of two identical and inverted copies of a 1258 bp-long IS*600* element in the *tra* region (positions 72898- 74161 and 95672- 94409, Acc. No. CP011417) in pINV_EF432_. The same region was lacking in pINV of 152661 (pINV_152661_), but remnants of IS*600* elements (1121/1199 nucleotidic identity, 46 gaps) were found in the corresponding positions (Figure 4).

**Table 1:** Analysis of the presence of conjugation-related genetic elements in the closed sequences of the plasmids of the O96:H19 strains EF432 and 152661.

**Strain and plasmid**	**Feature encoded**	**Location**
EF432 pINV	*oriT* region	74839.74924 (–)
293826 bp	Relaxase	110756.114385 (+)
(Acc. No. CP011417)	T4CP	102213.104438 (+)
	T4SS gene cluster	74272.115151
EF432 pRES	Relaxase	01885.7155 (–)
47606 bp (Acc. No. CP011418)	T4CP	07155.9443 (–)
152661 pINV	Relaxase	146372.147019 (–)
266880 bp	T4CP	0148908.149147 (–)
(Acc. No. CP046677.1)	T4SS gene cluster	139934.155870
152661 pRES	Relaxase	018019.23289 (+)
48742 bp (Acc. No. CP046678.1)	T4CP	015758.18019 (+)

The same analysis was performed to compare the *tra* region of pINV_EF432_ with those partially found in the pINV of other four isolates. The results, reported in Supplementary Figure 4, showed a complete panel of *tra* genes only in the pINV of strain CNM-2113/13. The *traD* gene encoding the T4CP was instead absent from the remaining three plasmids. Additionally, the genes *traN* and *traI* were absent in the pINV sequence of strain SRR3578770. Finally, the presence of different short insertion elements in all the strains but CNM-2113/13 was observed (Supplementary Figure 4).

#### Transcription Assay of Pilin-Encoding Gene *traA* in EF432 Strain

A reverse transcriptase Real Time PCR expression assay was deployed to verify the transcription of *traA* gene, encoding the main component of the conjugative pilus, present in pINV_EF432_. The results showed that the *traA* gene was transcribed in both the growth conditions used (incubation at 30° and 37°C), even if at lower levels than the housekeeping *gapA* gene (Table 2). The efficiency results of the reactions targeting *traA* and the housekeeping gene *gapA*, used as control, were comparable.

**Table 2:** Results of the reverse transcription real time PCR expression assay for *traA* and *gapA* genes.

	**mean Ct**	**Std. Dev. Ct**	**mean Ct**	**Std. Dev. Ct**
	***traA***	***traA***	***gapA***	***gapA***
cDNA 30	28.71	0.21	22.06	0.42
cDNA 37	27.59	0.11	25.14	0.30
DNA 2,25 ng/reaction	18.57	0.27	21.26	0.26
DNA 2,25 × 10^−1^ ng/reaction	22.66	0.30	24.96	0.41
DNA 2,25 × 10^−2^ ng/reaction	25.42	0.12	28.26	0.28
DNA 2,25 × 10^−3^ ng/reaction	28.65	0.20	31.76	0.93

#### Conjugation Between Donor EF432 Strain and Recipient CSH26Nal *E. coli* K12 Strain

Conjugation experiments between donor strain EF432 and the recipient *E. coli* K12 strain CSH26 Nal^r^ were performed to assess the ability of the strain EF432 to transfer one or both the pINV and pRES plasmids to the recipient strain. No red transconjugant colonies were identified on TSA plates supplemented with nalidixic acid and Congo Red. PCR screening for the presence of *ipaH* gene in a selection of 200 colonies also resulted negative. This was in line with the lack of red colonies observed, as the presence of pINV plasmid is associated with red color on plates containing Congo Red (Sakai et al., 1986). Nevertheless, when the conjugation mixture was plated on LB media supplemented with streptomycin, one colony was detected, which was proven to be also resistant to sulphametoxazole and negative to *ipaH* gene in PCR, resembling a trans-conjugative colony of CSH26 Nal^r^ which got the accessory plasmid harboring the resistance genes to the two antimicrobials present in EF432 strain.

### Phylogenetic Analysis of EIEC O96:H19 Strains in Comparison With Reference EIEC and *Shigella* Strains

**Figure 5:**
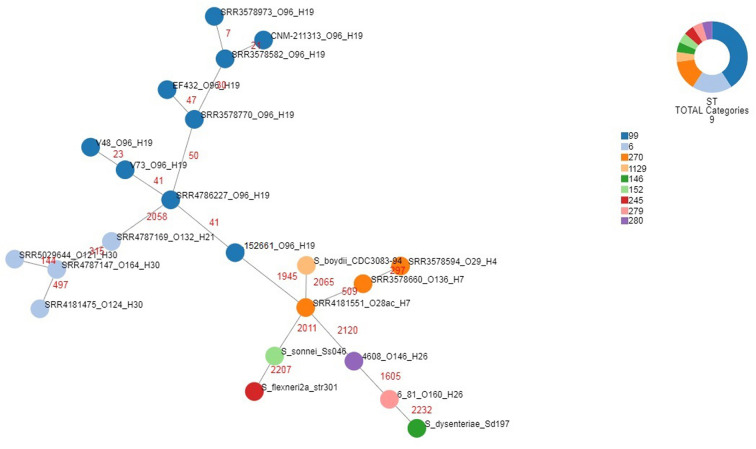
Phylogenomic analysis of EIEC and *Shigella* strains through cgMLST. For EIEC isolates, the strains identifiers include the strain name and the O and H antigens, each separated by underscores. For *Shigella* isolates, the strains identifiers comprise the species and the strain name. The different colors categorize the Sequence Types derived for the corresponding strains, as detailed in the legend. The numbers in red indicate the number of allelic differences identified for each link.

A whole genome comparison of the population of EIEC O96:H19 genomes studied was performed using cgMLST. The strains included were the EIEC strains isolated in Uruguay and EIEC strains belonging to the serotype O96:H19, including those isolated in the outbreaks occurred in Italy and the UK, the strain isolated in Spain and isolates described in a previous study on EIEC strains isolated from UK residents (Cowley et al., 2018). Moreover, EIEC strains representative of all the serotypes detected during the same study (Cowley et al., 2018) were included in the analysis for comparative purposes, as well as the 4608 and 6.81 EIEC reference strains and genomes from different *Shigella* species (Supplementary Table 1). The statistics of this analysis are reported in Supplementary Table 4, while the minimum spanning tree is shown in Figure 5. It is important to note that, with the only exception of the 6.81 reference EIEC strain whose published sequence showed the lowest quality, for all the other genomes alleles could be called for almost all the *loci* included in the cgMLST scheme used (Supplementary Table 4). The figure shows colors based on the detected Sequence Type. The O96:H19 strains, all belonging to ST-99, formed a homogeneous cluster. Two separate branches, one comprising only EIEC strains belonging to ST-6 and the second including all the other STs comprising all the *Shigella* and EIEC reference strains, segregated far apart from each other in terms of allelic distances and from the cluster of O96:H19 strains. Noteworthy, all *Shigella* strains occupied terminal positions, branching from one single EIEC strain typed as O28ac:H7 and showing the highest number of allelic distances from O96:H19 cluster.

## Discussion

The acquisition of genetic elements through horizontal gene transfer represents a major genetic mechanism driving the radiation of pathogenic *Escherichia coli* into different groups. Besides, the loss of genetic functions that are not required or could even have an adverse effect on the life of the bacterial pathogen offers additional advantages to certain pathogenic *E. coli* populations. Among diarrheagenic *E. coli*, Enteroinvasive *E. coli* (EIEC) are able to invade and replicate into the epithelial cells in the colon of the human host, with the same mechanism exerted by *Shigella* spp. (Pasqua et al., 2017). The key genetic event characterizing the evolution of EIEC and *Shigella* consisted in the acquisition of the pINV virulence plasmid, which harbors the majority of genes involved in the invasion mechanism, in combination with the accumulation of pathoadaptive mutations in anti-virulence *loci*, which has been extensively demonstrated either in EIEC or *Shigella* strains (Pasqua et al., 2017).

A novel EIEC O96:H19, emerging in Europe as a foodborne pathogen associated with outbreaks and sporadic cases, showed peculiar characteristics, such as lactose fermentation, motility, and lack of pathoadaptive mutations (Michelacci et al., 2016).

In the present paper we investigated the hypothesis of the emergence of such clone following an event of acquisition of the pINV plasmid by a non-pathogenic *E. coli* and studied its ability to exchange genetic material via conjugation. Additionally, we describe the characterization of EIEC O96:H19 strains isolated from two severe cases of infections occurred in Uruguay in 2014 (Peirano et al., 2018). The isolates from South America showed the absence in their genomes of pathoadaptive mutations and of chromosomal virulence genes usually found in EIEC and *Shigella*, while showed the presence of *gsp* genes, encoding a Type 2 Secretion System, also described in non-pathogenic *E. coli* (Stathopoulos et al., 2000). After the first description of EIEC O96:H19 (Michelacci et al., 2016), the whole genome sequences of other EIEC strains isolated in the UK were made available, including several O96:H19 isolates (Cowley et al., 2018). Four of these latter genomes were included in the present work and their comparison with the genomes of the other EIEC strains studied allowed to observe in one strain (Acc. No. SRR4786227) the lack of the complete “entry region”of the pINV virulence plasmids of EIEC and *Shigella*, which usually harbors the *ipa-mxi-spa locus* (Pasqua et al., 2017) (Figure 1 and Supplementary Table 2). The loss of such region had already been described in spontaneous avirulent isolates of *Shigella flexnerii* (Venkatesan et al., 2001). It is interesting to note that in both cases, the deletion also included the region encompassing *virA* and *icsA* genes, not physically linked to the “entry region,” but still pivotal for the pathogenesis of *Shigella* and EIEC. While it is not possible to determine if such regions were lost or had never been acquired, in another EIEC O96:H19 strain (Acc. No. SRR3578770) the region including *virA* and *icsA* was absent from the plasmid, in presence of a complete “entry region” (Figure 1 and Supplementary Table 2). This finding provides the first evidence, to the best of our knowledge, that the two regions can be acquired or lost in separate events. Moreover, the absence of *ospG* observed in two strains (Acc. No. SRR3578973 and SRR3578582), proving instead positive either for the “entry region” or the *virA-icsA* region, appeared in contrast with the previous hypothesis of co-acquisition of these *loci* (Buchrieser et al., 2000). Even if it is not possible to exclude that the absence of *ospG* gene in one of the strains could derive from a specific deletion event, its presence in the other, lacking the “entry region” and the *virA-icsA locus* (SRR4786227), suggests instead that the acquisition of *ospG locus* could have derived from an independent acquisition event.

Despite the absence of some EIEC virulence genes in many of the O96:H19 strains isolated in UK, it should be considered that they were all isolated from hospitalized or community cases of gastrointestinal disease (Cowley et al., 2018). In this respect, the reported presence of multiple transposons and insertion elements in pINV plasmids of EIEC and *Shigella* (Pasqua et al., 2017), together with previous reports of spontaneous loss of the “entry region” from pINV plasmids resulting in avirulent strains (Venkatesan et al., 2001), suggest that the loss of such virulence region in SRR4786227 could have occurred during isolation and subculturing of the strain.

The presence of the two virulence genes *capU* and *lpfA* in the EIEC O96:H19 strains, encoding an hexosyltransferase of Enteroaggregative *E coli* and rarely identified in *S. flexnerii* strains (Fujiyama et al., 2008; Ikumapayi et al., 2017) and the main subunit of the long polar fimbriae, frequently present in Shiga-toxin producing *E. coli* (STEC) and rarely identified in EIEC (Toma et al., 2006), respectively, may not be enough to explain the association of the strain lacking the “entry region” with gastrointestinal illness. Nevertheless, the conserved presence of these two genes suggests a role for their products in the virulence of EIEC O96:H19. Their function in the specific context of EIEC pathogenesis would necessitate further investigation.

The comparison of the virulence plasmids of the EIEC O96:H19 strains carried out in this study also highlighted the presence of a complete conjugation region in pINV of EF432 and CNM-2113/13 strains and a nearly complete region in other strains analyzed (namely SRR3578973, SRR3578770, and SRR3578582) (Figures 1, 2, 4 and Supplementary Figure 4). This region is completely lacking in pINV from reference EIEC and *Shigella* strains (Pasqua et al., 2017). The production of the completely closed sequences of the chromosomes and the plasmids of the strains EF432 and 152661 allowed a deeper investigation of the region involved in conjugation both in the pINV and in the accessory plasmid harboring resistance genes (pRES) found in the two strains (Figures 2, 3, Supplementary Figures 2, 3 and Table 1). This analysis confirmed the presence of the whole set of *loci* involved in conjugation only in pINV from the EF432 strain, supporting the hypothesis that this plasmid may be capable of transferring to other *E. coli* strains through conjugation. The observation of active transcription of the *traA* pilin-coding gene through Real Time PCR and the result of conjugation experiments suggesting the transfer of the accessory pRES plasmid to a recipient strain strengthened such a hypothesis. As a matter of fact, the pRES plasmid of EF432 carried the genes conferring resistance to streptomycin and sulfametoxazole, which could have both been transferred to the *E. coli* K12 CSH26 Nal^r^ used as recipient in the conjugation experiments. On the other hand, the pRES of this strain was negative for the *tra* genes involved in conjugation and, as such, could have been moved by exploiting an *in trans*-encoded conjugation machinery, which was, in our system, harbored on the pINV_EF432_. The lack of identification of CSH26 Nal^r^ transconjugant strains which acquired the pINV_EF432_ could be explained by the very low conjugation frequency (<10^−7^) previously reported for pINV plasmids of *Shigella* strains (Sansonetti et al., 1981), in association with the lack of a selective marker on pINV_EF432_. Further work involving different strategies such as tagging the plasmid with the insertion of an antimicrobial resistance gene cassette would ease the recovery of transconjugant clones confirming its capacity of self-mobilization through conjugation.

Altogether, these results are strongly indicative of the presence of a functional conjugation system at least in one O96:H19 EIEC strain, EF432. A similar system could also be present in the genome of the strain CNM-2113/13. Unfortunately, the sequence of this strain was not available as a closed genome and the fragmented contigs did not allow to completely characterize the *tra locus* on the plasmid (Supplementary Figure 4). The finding of the identification of two inverted copies of IS*600*, belonging to IS*3* family, at the two sides of the region of pINV_EF432_ encoding conjugative elements and the presence of remnants of a similar IS*600* sequence in the corresponding region of the pINV_152261_ (Figure 4) suggested that a recombination event between these two copies could have led to the deletion of the DNA stretch of about 23 kbp, resulting in a plasmid with a defective conjugation region in the latter strain. This mechanism could be part of the stabilization process of the pINV in EIEC O96:H19, resulting in progressive loss of conjugation ability, as reported for *Shigella* species (Johnson and Nolan, 2009). Nevertheless, the identification of other insertion elements and of inversions in the *tra* region of other O96:H19 strains analyzed suggests that multiple events could contribute to the inactivation of the *locus* and the resulting stabilization of the plasmid. Similarly, the observation of a mosaic virulence genes asset in the pINV of several EIEC O96:H19, together with a variable pattern in the presence of the conjugation region, supports the recent emergence of the EIEC O96:H19 clone and could be related with the activation of mobile genetic elements on the plasmid.

In order to visualize the phylogenetic relationships among the EIEC O96:H19 strains, an analysis was conducted using the core genome Multi Locus Sequence Typing, including reference EIEC and *Shigella* strains for comparison (Figure 5). A low number of allelic differences was observed among the different EIEC O96:H19 strains, which confirmed their recent evolution. This was also supported by the low variability in the chromosome structure as observed in the whole genome comparison performed by BRIG analysis (Supplementary Figure 1) and by the identification of prophages in the majority of the differing regions.

The apical positions occupied by all the *Shigella* strains in the minimum spanning tree, which shows these strains branching from typical EIEC strains belonging to Sequence Types 270, 279 and 280 (Figure 5), support the reclassification of *Shigella* into the *E. coli* species (Pettengill et al., 2015b) and are in line with the high specialization of such strains for living as intracellular pathogens. It is also interesting to note that all the EIEC strains belonging to ST-6 formed a separate branch, supporting the previous hypothesis of a separate evolution for this EIEC clone (Michelacci et al., 2016).

Altogether, the results of this study demonstrate that the circulation of the highly virulent O96:H19 EIEC clone is not restricted to Europe and provide evidences for its recent emergence showing still active evolution mechanisms.

Noteworthy, the retention by some of the O96:H19 EIEC strains of the genetic *locus* encoding a functional conjugation machinery adds strong evidence to the hypothesis of the emergence of novel EIEC clones through the acquisition of the pINV plasmid by commensal or environmental *E. coli* strains via conjugation. As a matter of fact, EIEC O96:H19 display, besides the presence of the pINV plasmid, good growing abilities and capacity of mobilization outside the cells (Michelacci et al., 2016).

It cannot be excluded that such a clone could have been circulating even before its first description during the 2012 outbreak (Escher et al., 2014), and that it remained undetected due to its peculiar characteristics. As a matter of fact, in routine diagnosis, the identification of EIEC and *Shigella* usually relies on the isolation of colonies lacking lactose fermentation, lysine decarboxylase activity and motility.

Notably, a foodborne outbreak caused by a novel O8:H19 EIEC clone was also reported in North Carolina in 2018, representing the first confirmed outbreak of EIEC infections in the United States in 47 years, and the first report of EIEC serotype O8:H19 (Herzig et al., 2019), confirming the importance of preparedness in the detection of the EIEC pathotype. Further work should be carried out to explore the possibility that other EIEC types, including the mentioned O8:H19, may possess hybrid characteristics encompassing the ability of striving outside a eukaryotic cell and the presence of pINV, thus demonstrating the evolutionary pathway connecting the *E. coli* genomic continuum and *Shigella* strains.

## Data Availability Statement

The datasets generated and used for this study can be found in the EMBL-European Nucleotide Archive Study with Acc. No. PRJEB35723 and at NCBI Bioproject with Acc. No. PRJNA315192. All the details about the Acc. No. of each sequencing run and of additional samples included in the study are listed in Supplementary Table 1.

## Author Contributions

VM conceived the experimental design and drafted the manuscript. RT contributed in the scientific discussion and particularly in the design of the conjugation and Real Time PCR experiments. AD'A performed the bioinformatics analyses on ARIES webserver. SA and AB prepared and checked the bacterial cultures and performed the Real Time PCR experiments. AK installed the ARIES server for the bioinformatic analyses and installed the tools for the data analysis. GP contributed in the discussion and provided the CSH26Nal strain for conjugation. DG and CJ took care of the Nanopore sequencing and data analysis at PHE and provided the strain from the United Kingdom, TC, AS, AN, FS, and GV provided and characterized the strains from Uruguay (in detail TC and AS made the initial characterization. GV and FS made the complete identification, including detection of the *ipaH* gene. AN performed the serotyping of both isolates). SM contributed in the scientific discussion and thoroughly revised the manuscript. Finally, all the authors approved the manuscript to be published.

## Conflict of Interest

The authors declare that the research was conducted in the absence of any commercial or financial relationships that could be construed as a potential conflict of interest.

## Footnotes

^1^*Porechop*. https://github.com/rrwick/Porechop

^2^*Filtlong*. https://github.com/rrwick/Filtlong

## Supplementary Material

Supplementary Tables 1, 2, 3 and 4 include, respectively: the list of strains used in this study, with accession numbers; virulotyping results of O96:H19 EIEC strains; regions of difference identified among chromosomes of O96:H19 strains; and the statistics of cgMLST analysis.

Supplementary Figures 1, 2, 3 and 4 illustrate, respectively: BRIG alignment among chromosomes of O96:H19 strains; genetic map of pRES from EF432; genetic map of pRES from 152661; alignment of *tra* regions in pINV of EF432 and of the strains harboring at least a part of the region.
