# Supplementary material for: Tracing Back the Evolutionary Route of Enteroinvasive Escherichia coli (EIEC) and Shigella Through the Example of the Highly Pathogenic O96:H19 EIEC Clone
Source: Front Cell Infect Microbiol. 2020 Jun 3;10:260. doi: 10.3389/fcimb.2020.00260 (PMC7283534; doi:10.3389/fcimb.2020.00260)
Supplement: Supplementary file 1 [file Table_1.DOCX]

**Supplementary Table 1.** List of strains used in this study, with accession numbers of the whole genome sequences available in the public repositories. NT: not typeble.

| **Strain** | **Classification** | **Origin** | **Year** | **ST** | **Serotype** | **Sequence read archive Accession Number** | **Completely assembled sequences Accession Number** |
| --- | --- | --- | --- | --- | --- | --- | --- |
| EF432 | EIEC | Italy | 2012 | 99 | O96:H19 |  | Chromosome: CP011416.1  pINV: CP011417.1  Second plasmid: CP011418.1 |
| 152661 | EIEC | United Kingdom | 2014 | 99 | O96:H19 | SRR4181492 | Chromosome: CP046676.1  pINV: CP046677.1  Second plasmid: CP046676.1 |
| V48 | EIEC | Uruguay | 2014 | 99 | O96:H19 | ERX3723112 |  |
| V73 | EIEC | Uruguay | 2014 | 99 | O96:H19 | ERX3723113 |  |
| CNM-2113/13 | EIEC | Spain | 2013 | 99 | O96:H19 | ERX3723114 |  |
| SRR4787147 | EIEC | United Kingdom | 2013 | 6 | O164:H30 | SRR4787147 |  |
| SRR4786227 | Unknown | United Kingdom | 2012 | 99 | O96:H19 | SRR4786227 |  |
| SRR4181551 | EIEC | United Kingdom (travel to Tanzany) | 2012 | 270 | O28ac:H7 | SRR4181551 |  |
| SRR3578973 | EIEC | United Kingdom (travel to Turkey) | 2014 | 99 | O96:H19 | SRR3578973 |  |
| SRR3578770 | EIEC | United Kingdom | 2015 | 99 | O96:H19 | SRR3578770 |  |
| SRR3578660 | EIEC | United Kingdom (travel to Indonesia) | 2014 | 270 | O136:H7 | SRR3578660 |  |
| SRR3578582 | EIEC | United Kingdom (travel to Turkey) | 2014 | 99 | O96:H19 | SRR3578582 |  |
| SRR3578594 | EIEC | United Kingdom | 2013 | 270 | O29:H4 | SRR3578594 |  |
| SRR4181475 | EIEC | United Kingdom | 2008 | 6 | O124:H30 | SRR4181475 |  |
| SRR4787169 | EIEC | United Kingdom | 2009 | 6 | O132:H21 | SRR4787169 |  |
| SRR5029644 | EIEC | United Kingdom (travel to Morocco) | 2005 | 6 | O121:H30 | SRR5029644 |  |
| 4608 | EIEC | USA | Before 1982 | 280 | O143:H26 | JTCO00000000 |  |
| 6.81 | EIEC | Africa | 1950 | 279 | O160:H26 | ERS756102 |  |
| Ss046 | *S*. *sonnei* | China | 1950 | 152 | ONT:H16 |  | NC_007384 |
| CDC3083-94 | *S. boydii* | USA | 2008 | 1129 | ONT:H45 |  | CP001063 |
| Sd197 | *S. dysenteriae* | China | 1950 | 146 | O148:H18 |  | NC_007606 |
| str. 301 | *S. flexnerii* 2a | China | 1984 | 245 | O13:H14 |  | NC_004337 |
