# Supplementary material for: Tracing Back the Evolutionary Route of Enteroinvasive Escherichia coli (EIEC) and Shigella Through the Example of the Highly Pathogenic O96:H19 EIEC Clone
Source: Front Cell Infect Microbiol. 2020 Jun 3;10:260. doi: 10.3389/fcimb.2020.00260 (PMC7283534; doi:10.3389/fcimb.2020.00260)
Supplement: Supplementary file 2 [file Table_2.DOCX]

**Supplementary Table 2.** Identification of plasmid-borne and chromosomally encoded virulence genes in the genomes of the O96:H19 EIEC strains analyzed in this study. T3SS: Type Three Secretion System

| ***Location in the reference genomes*** | **Gene** | **Gene function** | **Strain** | | | | | | | | |
| --- | --- | --- | --- | --- | --- | --- | --- | --- | --- | --- | --- |
|  |  |  | **EF432** | **CNM-2113/13** | **152661** | **V48** | **V73** | **SRR4786227** | **SRR3578973** | **SRR3578770** | **SRR3578582** |
| ***Plasmid*** | *icsA* | autotransporter, actin tail assembly protein IcsA/VirG | + | + | + | + | + | - | + | - | + |
|  | *icsB* | T3SS effector IcsB | + | + | + | + | + | - | + | + | + |
|  | *icsP* | outer membrane protease, involved in IcsA cleavage | + | + | + | + | + | + | + | + | + |
|  | *ipaA* | T3SS effector IpaA, vinculin binding protein | + | + | + | + | + | - | + | + | + |
|  | *ipaB* | T3SS hydrophilic translocator, pore protein IpaB | + | + | + | + | + | - | + | + | + |
|  | *ipaC* | T3SS hydrophilic translocator, pore protein IpaC | + | + | + | + | + | - | + | + | + |
|  | *ipaD* | T3SS hydrophilic translocator, needle tip protein IpaD | + | + | + | + | + | - | + | + | + |
|  | *ipaH7.8* | T3SS effector ipaH7.8, E3 ubiquitin ligase | + | + | + | + | + | + | + | + | + |
|  | *ipaH9.8* | T3SS effector ipaH9.8, E3 ubiquitin ligase | + | + | + | + | + | + | + | + | + |
|  | *ipgA* | chaperone for IcsB | + | + | + | + | + | - | + | + | + |
|  | *ipgC* | chaperone for IpaB and IpaC | + | + | + | + | + | - | + | + | + |
|  | *ipgD* | T3SS effector IpgD, phosphoinositide 4-phosphatase | + | + | + | + | + | - | + | + | + |
|  | *ipgE* | chaperone for IpgD | + | + | + | + | + | - | + | + | + |
|  | *ipgF* | T3SS protein IpgF | + | + | + | + | + | - | + | + | + |
|  | *mxiA* | T3SS major export apparatus protein MxiA | + | + | + | + | + | - | + | + | + |
|  | *mxiC* | T3SS gatekeeper MxiD | + | + | + | + | + | - | + | + | + |
|  | *mxiD* | T3SS secretin MxiD | + | + | + | + | + | - | + | + | + |
|  | *mxiE* | T3SS regulatory protein MxiE | + | + | + | + | + | - | + | + | + |
|  | *mxiG* | T3SS outer MS ring protein MixG | + | + | + | + | + | - | + | + | + |
|  | *mxiH* | T3SS needle filament protein MxiH | + | + | + | + | + | - | + | + | + |
|  | *mxiI* | T3SS inner rod protein MxiI | + | + | + | + | + | - | + | + | + |
|  | *mxiJ* | T3SS inner MS ring protein MxiJ | + | + | + | + | + | - | + | + | + |
|  | *mxiK* | T3SS accessory cytosolic protein MxiK | + | + | + | + | + | - | + | + | + |
|  | *mxiL* | T3SS protein MxiL | + | + | + | + | + | - | + | + | + |
|  | *mxiM* | T3SS pilotin MxiM | + | + | + | + | + | - | + | + | + |
|  | *mxiN* | T3SS stator MxiN | + | + | + | + | + | - | + | + | + |
|  | *ospG* | T3SS effector OspG, kinase | + | + | + | + | + | + | - | + | - |
|  | *senB* | enterotoxin | + | + | + | + | + | + | + | + | + |
|  | *spa9* | Mxi-Spa secretion machinery protein Spa9 | + | + | + | + | + | - | + | + | + |
|  | *spa13* | Mxi-Spa secretion machinery protein Spa13 | + | + | + | + | + | - | + | + | + |
|  | *spa15* | Mxi-Spa secretion machinery protein Spa15 | + | + | + | + | + | - | + | + | + |
|  | *spa24* | Mxi-Spa secretion machinery protein Spa24 | + | + | + | + | + | - | + | + | + |
|  | *spa29* | Mxi-Spa secretion machinery protein Spa29 | + | + | + | + | + | - | + | + | + |
|  | *spa32* | Mxi-Spa secretion machinery protein Spa32 | + | + | + | + | + | - | + | + | + |
|  | *spa33* | Mxi-Spa secretion machinery protein Spa33 | + | + | + | + | + | - | + | + | + |
|  | *spa40* | Mxi-Spa secretion machinery protein Spa40 | + | + | + | + | + | - | + | + | + |
|  | *spa47* | Mxi-Spa secretion machinery protein Spa47, putative ATPase | + | + | + | + | + | - | + | + | + |
|  | *virA* | T3SS effector VirA, GTPase-activating protein (GAP) | + | + | + | + | + | - | + | - | + |
|  | *virB* | transcriptional activator required for tanscription of the ipa, mxi, and spa operons | + | + | + | + | + | - | + | + | + |
|  | *virF* | transcriptional activator of virulence loci | + | + | + | + | + | + | + | + | + |
| ***Chromosome*** | *gspC* | general secretion pathway protein C | + | + | + | + | + | + | + | + | + |
|  | *gspD* | general secretion pathway protein D | + | + | + | + | + | + | + | + | + |
|  | *gspE* | general secretion pathway protein E | + | + | + | + | + | + | + | + | + |
|  | *gspF* | general secretion pathway protein F | + | + | + | + | + | + | + | + | + |
|  | *gspG* | general secretion pathway protein G | + | + | + | + | + | + | + | + | + |
|  | *gspH* | general secretion pathway protein H | + | + | + | + | + | + | + | + | + |
|  | *gspI* | general secretion pathway protein I | + | + | + | + | + | + | + | + | + |
|  | *gspJ* | general secretion pathway protein J | + | + | + | + | + | + | + | + | + |
|  | *gspK* | general secretion pathway protein K | + | + | + | + | + | + | + | + | + |
|  | *gspL* | general secretion pathway protein L | + | + | + | + | + | + | + | + | + |
|  | *gspM* | general secretion pathway protein M | + | + | + | + | + | + | + | + | + |
|  | *gtrA* | bactoprenol-linked glucose translocase/flippase | - | - | - | - | - | - | - | - | - |
|  | *gtrB* | bactoprenol glucosyl transferase | - | - | - | - | - | - | - | - | - |
|  | *gtrII* | glucosyl tranferase II | - | - | - | - | - | - | - | - | - |
|  | *iucA* | aerobactin synthesis protein IucA | - | - | - | - | - | - | - | - | - |
|  | *iucB* | aerobactin synthesis protein IucB | - | - | - | - | - | - | - | - | - |
|  | *iucC* | aerobactin synthesis protein IucC | - | - | - | - | - | - | - | - | - |
|  | *iutA* | aerobactin receptor IutA | - | - | - | - | - | - | - | - | - |
|  | *pic* | serine protease autotransporter Pic | - | - | - | - | - | - | - | - | - |
|  | *set1A* | Shigella enterotoxin 1 protein ShET1A | - | - | - | - | - | - | - | - | - |
|  | *set1B* | Shigella enterotoxin 1 protein ShET1B | - | - | - | - | - | - | - | - | - |
|  | *sigA* | serine protease autotransporter SigA | - | - | - | - | - | - | - | - | - |
|  | *stxA* | Shiga toxin subunit A; RNA-N-glycosidase; catalytic subunit | - | - | - | - | - | - | - | - | - |
|  | *stxB* | Shiga toxin subunit B; receptor binding subunit | - | - | - | - | - | - | - | - | - |
