# Supplementary material for: Tracing Back the Evolutionary Route of Enteroinvasive Escherichia coli (EIEC) and Shigella Through the Example of the Highly Pathogenic O96:H19 EIEC Clone
Source: Front Cell Infect Microbiol. 2020 Jun 3;10:260. doi: 10.3389/fcimb.2020.00260 (PMC7283534; doi:10.3389/fcimb.2020.00260)
Supplement: Supplementary file 3 [file Table_3.DOCX]

## Supplementary Table 3. List of regions of difference highlighted through BRIG comparison between the reference chromosome of EF432 strain and the chromosomes of the other O96:H19 analyzed in this study, including reference position, encoded features and regions length.

| **Position in pINV of EF432 (Acc. No. CP011416)** | **Features identified in annotation** | **Region length** |
| --- | --- | --- |
| 12700-38600 | DLP12 prophage | 25901 bp |
| 362726-408381 | DLP12/Rac/Qin prophage | 45656 bp |
| 543228-561435 | DLP12/Qin/Rac/e14 prophage | 18208 bp |
| 960626-967600 | Lambda/CP4-57 prophage | 6975 bp |
| 1652545-1665957 | YhdW/YhdX/YhdY/YhdZ ABC transporter coding genes; tRNAs; aroE (shikimate dehydrogenase); tsaC (protein involved in synthesis of threonylcarbamoyladenosine-modified tRNA); yrdD (putative DNA topoisomerase) | 13413 bp |
| 2159469-2185136 | rbSACBKR (ribose ABC transporter); hsrA (inner membrane protein); yieP (putative transcriptional regulator); rRNAs; tRNAs; hdfR (DNA-binding transcriptional dual regulator); yifB (putative ATP-dependent protease); ilvBMEDAYC (acetolactate synthase, enzymes of the isoleucine-valine biosynthetic pathway, regulator and isomeroreductase) | 25668 bp |
| 2251407-2269899 | tatABCD (protein export complex);  rfaH (transcriptional antiterminato); ubiD (3-octaprenyl-4-hydroxybenzoate carboxy-lyase monomer); fre (riboflavin reductase [NAD(P)H] / FMN reductase); fadAB (fatty acid oxidation complex); pepQ (proline dipeptidase); yigZ (putative elongation factor); trkH (K+ transporter); hemG (protoporphyrinogen oxidase); tRNAs; rRNAs | 18493 bp |
| 2403298-2408594 | rRNAs; tRNAs | 5297 bp |
| 2439842-2449467 | hyfR (DNA-binding transcriptional activator); purD (phosphoribosylamine-glycine ligase); purH (AICAR transformylase / IMP cyclohydrolase); rRNAs; tRNAs | 9626 bp |
| 3151536- 3164233 | tRNAs; ORFs putatively encoding hypothetical proteins, transcriptional regulator, ABC transporter ATPase, ACP S-malonyltransferase; yagP (putative transcriptional regulator LYSR-type) | 12698 bp |
| 3687773-3699733 | DLP12/Qin/CPZ-55 prophage | 11961 bp |
| 4136005-4156105 | DLP12/Rac prophage | 20101 bp |
| 4699719-4702525 | DLP12 prophage | 2807 bp |
