# Supplementary material for: Tracing Back the Evolutionary Route of Enteroinvasive Escherichia coli (EIEC) and Shigella Through the Example of the Highly Pathogenic O96:H19 EIEC Clone
Source: Front Cell Infect Microbiol. 2020 Jun 3;10:260. doi: 10.3389/fcimb.2020.00260 (PMC7283534; doi:10.3389/fcimb.2020.00260)
Supplement: Supplementary file 4 [file Table_4.DOCX]

**Supplementary Table 4:** Statistics of the cgMLST analysis performed through chewBBACA**.**

For EIEC isolates, the strains identifiers include the strain name and the O and H antigens, each separated by underscores. For *Shigella* isolates, the strains identifiers comprise the species and the strain name. EXC: Exact match; INF: inferred new alleles using Prodigal CDS predictions; LNF: locus not found; PLOT: Possible Locus On the Tip; NIPH: Non-Informative Paralogous Hit; ALM: Alleles Larger than Mode; ASM: Alleles Smaller than Mode.

| **Genome** | **EXC** | **INF** | **LNF** | **PLOT** | **NIPH** | **ALM** | **ASM** |
| --- | --- | --- | --- | --- | --- | --- | --- |
| 4608_O146_H26 | 2329 | 0 | 20 | 3 | 1 | 4 | 3 |
| 6_81_O160_H26 | 979 | 394 | 636 | 14 | 2 | 15 | 320 |
| CNM-211313_O96_H19 | 2346 | 3 | 1 | 2 | 3 | 0 | 5 |
| EF432_O96_H19 | 2348 | 0 | 3 | 0 | 3 | 0 | 6 |
| 152661_O96_H19 | 2341 | 0 | 8 | 5 | 3 | 0 | 3 |
| S_boydii_CDC3083-94 | 2234 | 0 | 93 | 0 | 2 | 0 | 31 |
| S_dysenteriae_Sd197 | 2143 | 0 | 155 | 0 | 3 | 1 | 58 |
| S_flexneri2a_str301 | 2338 | 0 | 11 | 0 | 2 | 2 | 7 |
| S_sonnei_Ss046 | 2359 | 0 | 0 | 0 | 1 | 0 | 0 |
| SRR3578582_O96_H19 | 2352 | 0 | 1 | 2 | 3 | 0 | 2 |
| SRR3578594_O29_H4 | 2268 | 20 | 60 | 9 | 1 | 0 | 2 |
| SRR3578660_O136_H7 | 2308 | 1 | 40 | 7 | 1 | 0 | 3 |
| SRR3578770_O96_H19 | 2347 | 1 | 5 | 2 | 3 | 0 | 2 |
| SRR3578973_O96_H19 | 2350 | 3 | 1 | 1 | 3 | 0 | 2 |
| SRR4181475_O124_H30 | 2271 | 22 | 51 | 5 | 7 | 0 | 4 |
| SRR4181551_O28ac_H7 | 2300 | 0 | 43 | 11 | 2 | 0 | 4 |
| SRR4786227_O96_H19 | 2343 | 2 | 6 | 4 | 3 | 0 | 2 |
| SRR4787147_O164_H30 | 2327 | 2 | 18 | 7 | 2 | 0 | 4 |
| SRR4787169_O132_H21 | 2310 | 13 | 24 | 8 | 2 | 0 | 3 |
| SRR5029644_O121_H30 | 2312 | 5 | 32 | 6 | 2 | 0 | 3 |
| V48_O96_H19 | 2345 | 1 | 1 | 2 | 3 | 0 | 8 |
| V73_O96_H19 | 2345 | 3 | 2 | 1 | 3 | 0 | 6 |
