# Supplementary figures and images for: Tracing Back the Evolutionary Route of Enteroinvasive Escherichia coli (EIEC) and Shigella Through the Example of the Highly Pathogenic O96:H19 EIEC Clone
Source: Front Cell Infect Microbiol. 2020 Jun 3;10:260. doi: 10.3389/fcimb.2020.00260 (PMC7283534; doi:10.3389/fcimb.2020.00260)

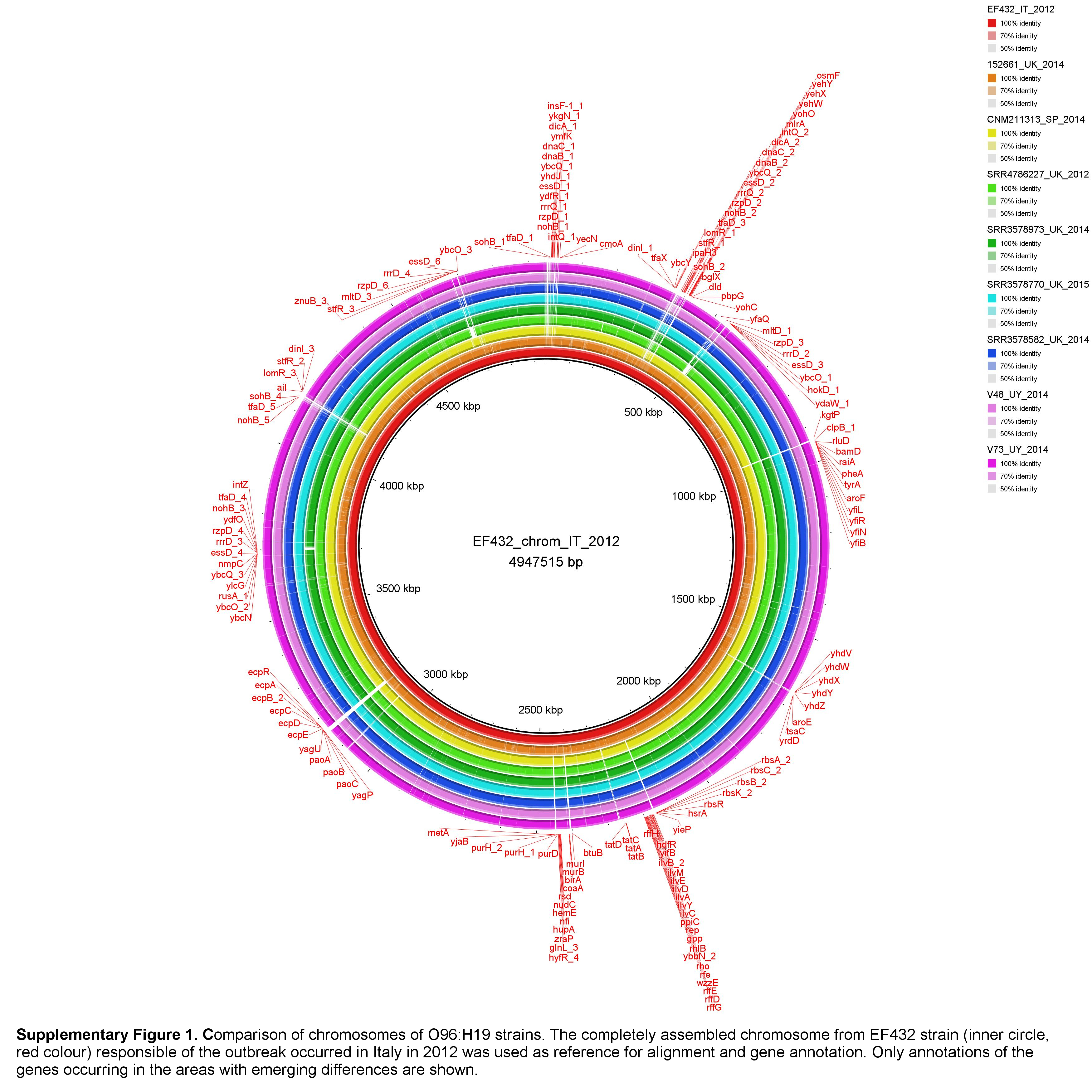

Supplement: Supplementary file 5 [file Image_1.JPEG]

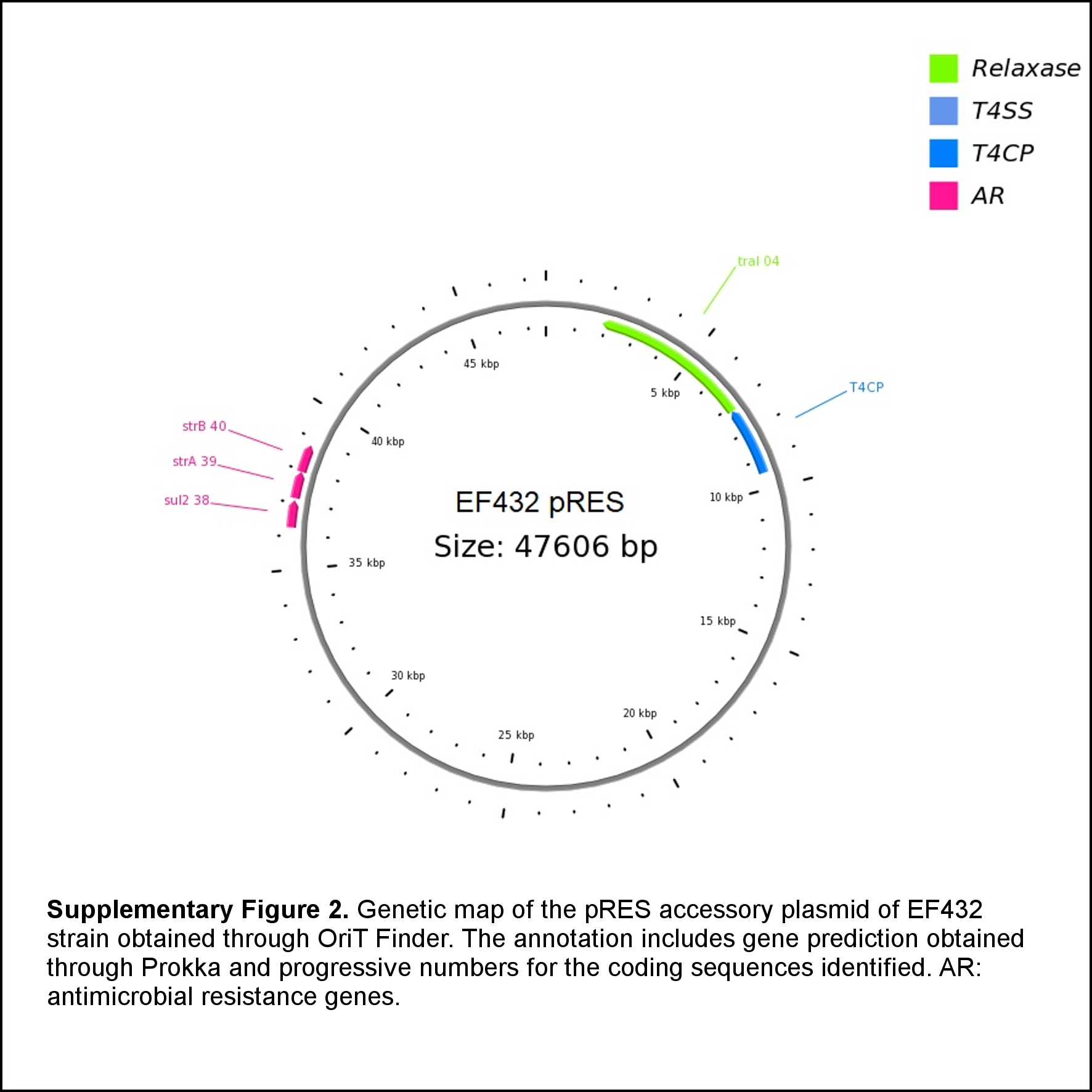

Supplement: Supplementary file 6 [file Image_2.JPEG]

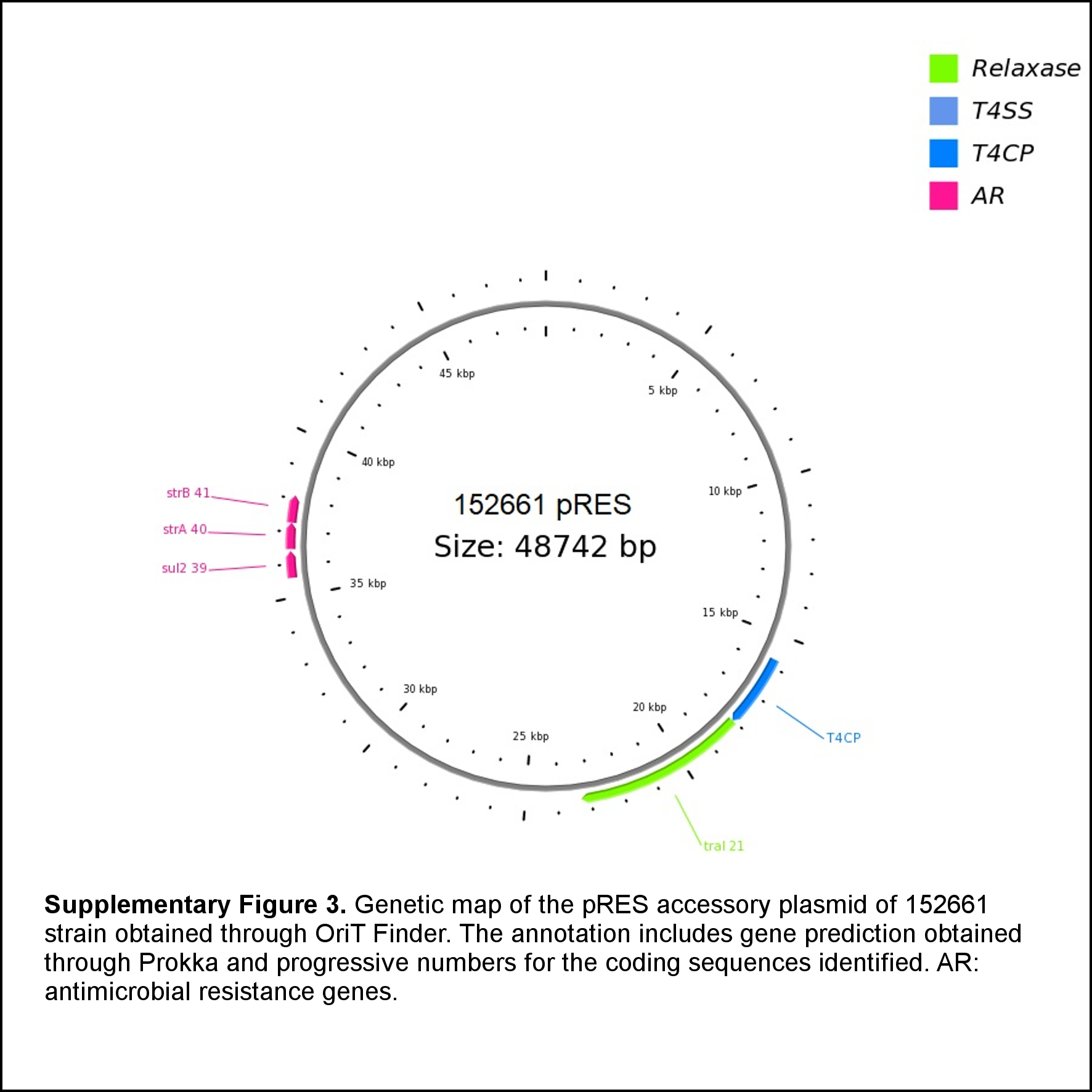

Supplement: Supplementary file 7 [file Image_3.JPEG]

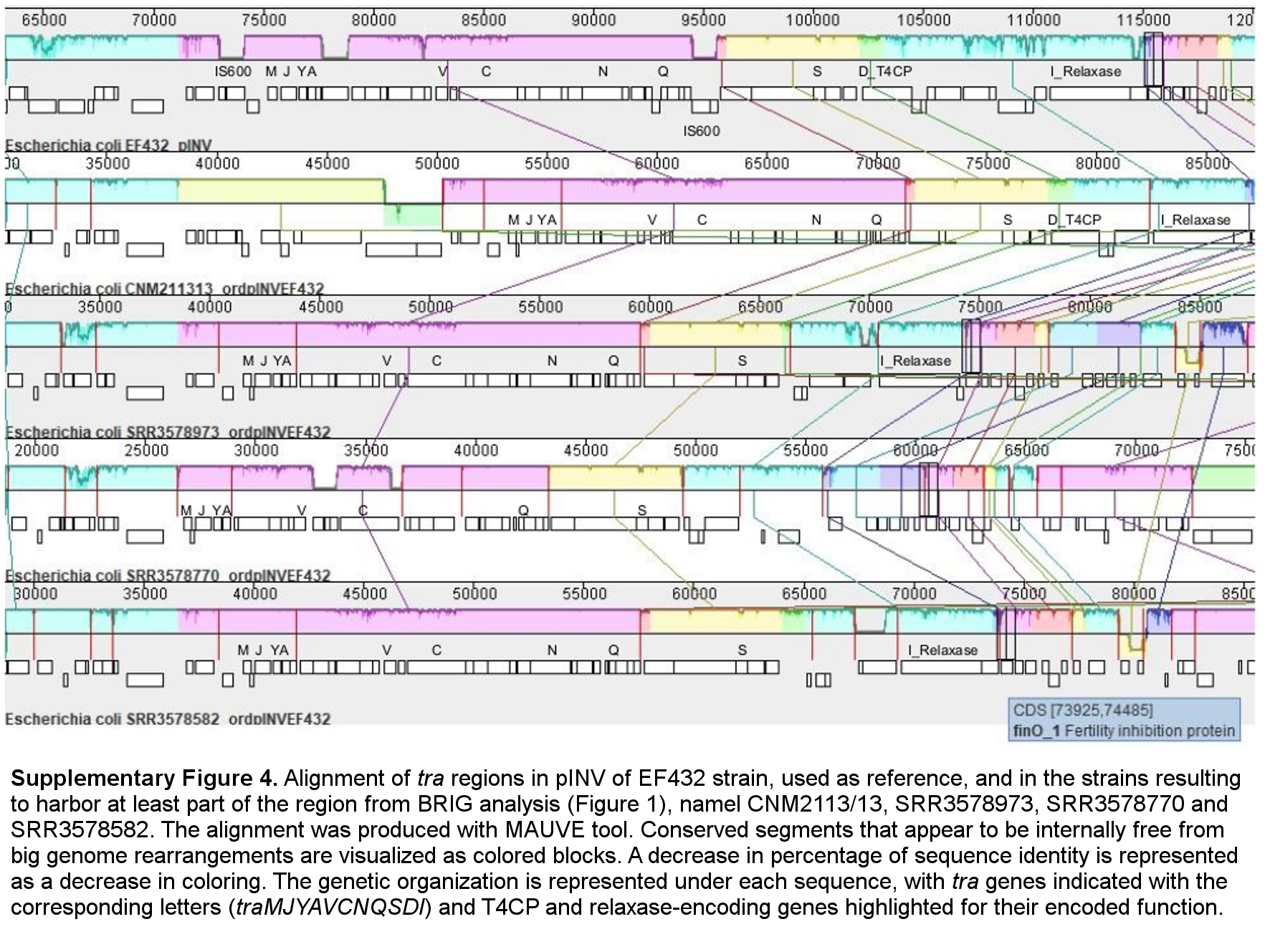

Supplement: Supplementary file 8 [file Image_4.JPEG]
